# Pan-transcriptome assembly combined with multiple association analysis provides new insights into the regulatory network of specialized metabolites in the tea plant *Camellia sinensis*

**DOI:** 10.1093/hr/uhac100

**Published:** 2022-07-02

**Authors:** Weilong Kong, Mengwei Jiang, Yibin Wang, Shuai Chen, Shengcheng Zhang, Wenlong Lei, Kun Chai, Pengjie Wang, Renyi Liu, Xingtan Zhang

**Affiliations:** Shenzhen Branch, Guangdong Laboratory for Lingnan Modern Agriculture, Genome Analysis Laboratory of the Ministry of Agriculture, Agricultural Genomics Institute at Shenzhen, Chinese Academy of Agricultural Sciences, Shenzhen 518120, China; Center for Genomics and Biotechnology, Haixia Institute of Science and Technology, Fujian Agriculture and Forestry University, Fuzhou 350002, China; Shenzhen Branch, Guangdong Laboratory for Lingnan Modern Agriculture, Genome Analysis Laboratory of the Ministry of Agriculture, Agricultural Genomics Institute at Shenzhen, Chinese Academy of Agricultural Sciences, Shenzhen 518120, China; Shenzhen Branch, Guangdong Laboratory for Lingnan Modern Agriculture, Genome Analysis Laboratory of the Ministry of Agriculture, Agricultural Genomics Institute at Shenzhen, Chinese Academy of Agricultural Sciences, Shenzhen 518120, China; Shenzhen Branch, Guangdong Laboratory for Lingnan Modern Agriculture, Genome Analysis Laboratory of the Ministry of Agriculture, Agricultural Genomics Institute at Shenzhen, Chinese Academy of Agricultural Sciences, Shenzhen 518120, China; Center for Genomics and Biotechnology, Haixia Institute of Science and Technology, Fujian Agriculture and Forestry University, Fuzhou 350002, China; Center for Genomics and Biotechnology, Haixia Institute of Science and Technology, Fujian Agriculture and Forestry University, Fuzhou 350002, China; Shenzhen Branch, Guangdong Laboratory for Lingnan Modern Agriculture, Genome Analysis Laboratory of the Ministry of Agriculture, Agricultural Genomics Institute at Shenzhen, Chinese Academy of Agricultural Sciences, Shenzhen 518120, China; Center for Agroforestry Mega Data Science, Haixia Institute of Science and Technology, Fujian Agriculture and Forestry University, Fuzhou 350002, China; Shenzhen Branch, Guangdong Laboratory for Lingnan Modern Agriculture, Genome Analysis Laboratory of the Ministry of Agriculture, Agricultural Genomics Institute at Shenzhen, Chinese Academy of Agricultural Sciences, Shenzhen 518120, China; Center for Genomics and Biotechnology, Haixia Institute of Science and Technology, Fujian Agriculture and Forestry University, Fuzhou 350002, China

## Abstract

Specialized metabolites not only play important roles in biotic and abiotic stress adaptation of tea plants (*Camellia sinensis* (L.) O. Kuntze) but also contribute to the unique flavor of tea, the most important nonalcoholic beverage. However, the molecular networks and major genes that regulate specialized metabolites in tea plants are not well understood. Here, we constructed a population-level pan-transcriptome of the tea plant leaf using second-leaf transcriptome data from 134 accessions to investigate global expression differences in the population, expression presence or absence variations (ePAVs), and differentially expressed genes (DEGs) between pure *Camellia sinensis* var. *assamica* (CSA) and pure *Camellia sinensis* var. *sinensis* (CSS) accessions. Next, we used a genome-wide association study, a quantitative trait transcript study, and a transcriptome-wide association study to integrate genotypes, accumulation levels of specialized metabolites, and expression levels of pan-transcriptome genes to identify candidate regulatory genes for flavor-related metabolites and to construct a regulatory network for specialized metabolites in tea plants. The pan-transcriptome contains 30 482 expressed genes, 4940 and 5506 of which were newly annotated from a *de novo* transcriptome assembly without a reference and a genome reference-based assembly, respectively. DEGs and ePAVs indicated that CSA and CSS were clearly differentiated at the population transcriptome level, and they were closely related to abiotic tolerance and secondary metabolite synthesis phenotypes of CSA and CSS based on gene annotations. The regulatory network contained 212 specialized metabolites, 3843 candidate genes, and 3407 eQTLs, highlighting many pleiotropic candidate genes, candidate gene-rich eQTLs, and potential regulators of specialized metabolites. These included important transcription factors in the AP2/ERF-ERF, MYB, WD40, and bHLH families. *CsTGY14G0001296*, an ortholog of *AtANS,* appeared to be directly related to variation in proanthocyanins in the tea plant population, and the *CsTGY11G0002074* gene encoding F3′5′H was found to contribute to the biased distribution of catechins between pure CSAs and pure CSSs. Together, these results provide a new understanding of the metabolite diversity in tea plants and offer new insights for more effective breeding of better-flavored tea varieties.

## Teaser

• A population-level pan-transcriptome of tea plant leaves was constructed.

• Multi-omics analysis revealed novel candidate genes for the regulation of specialized metabolites in tea plants.

• Candidate gene-rich eQTLs and key transcription factors were highlighted based on a global specialized metabolite network.

• *CsTGY14G0001296* (*ANS*) may be a key gene that causes variation in the content of many proanthocyanins among tea plants.

• *CsTGY11G0002074* (*F3′5′H*) may be responsible for the biased distribution of catechins between pure CSS and pure CSA accessions

## Introduction

The tea plant *Camellia sinensis* (L.) O. Kuntze is an important beverage crop worldwide, and the leaves of this crop are the source of the popular nonalcoholic beverage known as “tea” [1–3]. Various specialized metabolites in tea leaves make a major contribution to the health benefits of tea beverages, as well as to their unique taste and fragrance [4]. Flavonoids, theanine, and caffeine are the three major characteristic specialized metabolites that determine the rich flavors, fresh taste, and health benefits of tea [3, 5]. For instance, theanine and caffeine are important causes of the fresh and bitter taste of tea, respectively [6]. Flavonol glycosides contribute to the astringency of tea and can synergize with caffeine to enhance the bitterness induced by caffeine [7]. Relative to other plant species, tea also includes higher levels of the same or specialized aroma compounds and their glycosides, which increase the olfactory comfort of tea [8]. Therefore, understanding the genetic mechanisms of the synthesis of these metabolites in tea plants is important and will provide essential information for the regulation and improvement of tea quality.

The main pathways and structural genes that encode key enzymes associated with the most important specialized metabolites need to be fully elucidated. However, only catechins, caffeine, and theanine are relatively well understood to date [9, 10]. Catechins were previously reported to be synthesized through the phenylpropanoid and flavonoid pathways [11, 12]. The biosynthesis of caffeine requires three main methylation steps, which sequentially convert xanthine into 7-methylxanthine, theobromine, and finally caffeine [13]. Theanine biosynthesis starts from glutamine and pyruvate and depends on the enzymatic processes of TS (theanine synthetase), GS (glutamine synthetase), GLS (glutaminase), ALT (alanine aminotransferase), and ADC (arginine decarboxylase) [14, 15]. However, the synthesis of other specialized tea metabolites is not yet understood, and key regulatory factors (such as transcription factors) that control the synthesis of well-known catechins, caffeine, and theanine are still poorly understood.

With the popularity of high-throughput transcriptomics and metabolomics, “omics” approaches have been introduced for exploration of the underlying regulatory networks of tea plant specialized metabolites; such methods include comparative transcriptome analysis, comparative metabolomics analysis, and genome-wide association studies (GWASs) [1, 3]. Yu et al. (2020) reported that *Camellia sinensis* var*. assamica* (CSA) accessions accumulate higher levels of multiple flavonoid compounds, including flavanols, flavonol mono- and diglycosides, procyanidin dimers, and phenolic acids, than *Camellia sinensis* var*. sinensis* (CSS). Nonetheless, the major loci and genes that control these differences in specialized metabolites between CSA and CSS remain unknown [1]. Several recent studies have used a GWAS approach to explore major genes associated with several important specialized metabolites. Hazra et al. (2020) conducted a GWAS analysis of 23 Darjeeling tea cultivars and identified 12 SNPs related to epigallocatechin gallate (EGCG), 8 SNPs related to flavonoids, 8 SNPs related to flavor, and 3 SNPs related to phenolics [16]. Zhang et al. (2020) conducted a GWAS on the catechin trait of 176 ancient tea plant accessions and revealed that the *CsANR*, *CsF3′5′H*, and *CsMYB5* genes may affect tea plant catechin content [17]. Another GWAS identified 307 SNP markers related to theanine, caffeine, and catechins [5]. However, molecular studies of tea plant specialized metabolites are lagging substantially behind those of other crops such as maize [18, 19], rice [20, 21], and tomato [22].

The genome changes dynamically at the species level, and the genome of a single individual can reflect only a small portion of the diversity of the population-wide genome [23, 24]. Many earlier studies have shown that the use of QTL mapping and GWAS analysis based on a reference genome to explore relationships between molecular markers (SNPs or indels) and phenotypes can explain only part of the phenotypic variation [25, 26]. Presence/absence variations (PAVs) and copy number variations (CNVs) of expressed genes may make a great contribution to the diversity and plasticity of crop phenotypes and may partially explain the missed heredity in GWAS analysis or QTL mapping of genetic variation [26–29]. However, it is difficult to find important trait-related ePAVs and CNVs based on a single reference genome, and this problem prompted the proposal of the pan-genome concept [30]. As a result, extensive pan-genome research has been performed in maize [31], rice [32, 33], soybean [34, 35], and other crops [36–39]. However, constructing the pan-genome of a plant species is expensive and labor intensive, especially for species with complex and large genomes, such as the tea plant. The plant pan-transcriptome can be an effective alternative because of its relatively low cost and ability to detect genome and gene expression variations at the same time [28, 40]. For example, pan-transcriptomes of maize, tomato, barley, alfalfa, and potato have provided novel insights into genome complexity and quantitative trait variations [25, 28, 40–42]. However, to date, neither pan-genome nor pan-transcriptome analysis of the tea plant has been reported. Here, we used a pan-transcriptome approach to analyze genetic and expression variations in a tea population and multiple association analysis of specialized metabolites to provide new insights into the regulation of specialized metabolites in tea.

With the development of high-throughput sequencing technology and untargeted metabolomics, extensive transcriptomic and metabolomic data have been released for many tea varieties, allowing us to use multi-omics methods to integrate genotypes, specialized metabolite accumulation, and transcriptomes to systematically analyze the regulatory network of specialized metabolites [1]. Here, we assembled a pan-transcriptome of tea leaves by integrating a population-level transcriptome assembly based on a high-quality ‘Tieguanyin’ (TGY) reference genome and a *de novo* assembly with reads that could not be mapped to the TGY genome. In addition, we characterized the gene expression patterns of 134 tea plant accessions and identified ePAVs and DEGs between pure CSA and pure CSS accessions. We also established a series of specialized metabolite–gene–eQTL relationships by integrating the results of genome-wide association studies, quantitative trait transcript analyses, and transcriptome-wide association studies. Finally, we obtained a global tea plant regulatory network of specialized metabolites and revealed several key genes for specialized metabolite production through gene correlation analysis. Our results will help improve our understanding of the molecular mechanisms that control tea plant specialized metabolites and contribute to the improvement of tea flavor.

## Results

### Pan-transcriptome of the tea plant leaf

Transcriptome analysis based on genes annotated in a single reference genome is not sufficient to systematically explore genetic differences that underlie crucial traits at the tea plant population level because a single reference genome lacks many subspecies and variety genes that are strongly associated with specialized metabolites and biological pathways [23, 28]. To construct the pan-transcriptome of the tea plant leaf, we downloaded transcriptome sequencing data (PRJNA562973) for second-leaf samples from 136 accessions (128 cultivars) [1], divided them into three groups based on a phylogenetic tree, and performed a population-level transcript assembly for each group based on a reference genome-based and *de novo* assembly strategy (see the pipeline for pan-transcriptome assembly in Figure S1).

**Figure 1:**
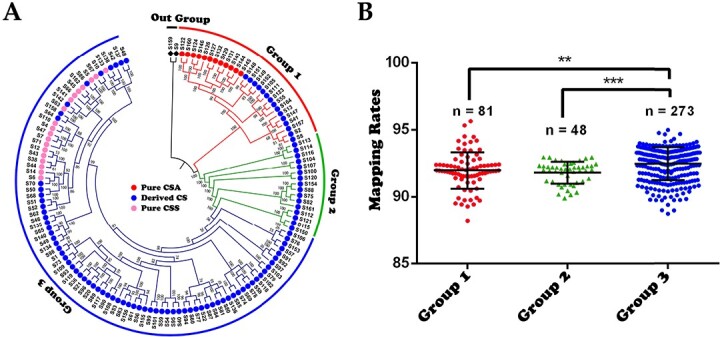
Genomic divergence of tea plant accessions. A. Maximum likelihood-based phylogenetic tree of 136 tea plant accessions built with the GTR model and 1000 bootstrap replicates in IQ-TREE. The tree was rooted to S159 (a close relative of the tea plant) and S9 (“Chaoyang”, derived from a wild tea plant). The red, green, and blue arcs and branches represent Groups 1, 2, and 3, respectively. B. Read mapping rates of the three groups. ^**^ and ^***^ represent p-values <0.01 and <0.001 (Student’s t-test).

RNA-sequencing data from 136 accessions (three biological replicates per accession) were mapped onto a high-quality genome of Tieguanyin (TGY) [43] with a mapping rate ranging from 88.22% to 95.63%, except for S159 (78.53%–83.42% for the wild tea plant accession Dali Yeshengcha), suggesting that the sequencing data were of good quality [1]. A total of 2 285 251 chromosome-anchored SNPs (by PLINK v1.9 with parameters maf < 0.05 and geno <0.3) were identified on 15 chromosomes and were further filtered to 643 296 SNPs based on SNP linkage relationships for maximum likelihood-based phylogenetic tree construction and population structure analysis. All 136 accessions were divided into three groups consistent with the previous group divisions of Yu et al. (2020) [1], although a subtle difference was that Yu et al.’s Group 3, 4, and 5 were merged into Group 3 because of its CSS genetic dominance and for ease of pan-transcriptome assembly (Figure 1A and Figure S2).

The results of the population structure analysis supported the phylogenetic tree-based group divisions. With K = 2, CSA and CSS were clearly highlighted, and there were some hybrid descendants of CSA and CSS in Group 1 and Group 3 corresponding to documented modern breeding events [1] (Figure S2). Here, we accepted the accessions in which the genetic background never segregated as pure CSA (12 in Group 1) and pure CSS (19 in Group 2) when K (the number of populations) was 2–5 (Figure S2). We found that pure CSA accessions or CSS accessions tended to cluster together in the maximum likelihood-based phylogenetic tree (Figure 1A and Figure S2). The remaining accessions were named Derived CS to reflect potential hybridization between CSA and CSS or potential genetic introgressions of closely related species (Figure S2 and Table S1).

As the next step, we assembled a population-level transcriptome based on the TGY reference genome. First, we mapped clean reads from each accession onto the TGY genome, and the average mapping rates of Groups 1, 2, and 3 were 91.97%, 91.80%, and 92.47%, respectively (Figure 1B). The mapping rates of Groups 1 and 2 were substantially lower than those of Group 3, suggesting that there may be genes in the Group 1 and 2 accessions that are not present in the TGY reference genome.

Next, we performed transcript assembly of each group with StringTie (see detailed pipeline in the **Materials and Methods** and Figure S1) and obtained a primary TGY-reference pan-transcriptome using taco-v0.7.3 to integrate the assembly results of the three groups. The pan-transcriptome contained 78 720 genes encoding 192 787 transcripts. All transcripts were quantified by calculating fragments per kilobase of transcript length per million reads (FPKM) using the primary pan-transcriptome as a reference. Then, transcripts with an average expression per accession (total FPKM/number of biological replicates) <1 FPKM were filtered out as low expressed genes. Finally, a TGY-reference pan-transcriptome containing 25 522 genes encoding 46 243 transcripts was obtained, including 20 016 TGY genes and 5506 newly annotated genes.

As the second step, 76.07 Gb, 45.92 Gb, and 193.89 Gb of unmapped reads from Groups 1, 2, and 3, respectively, were used for *de novo* transcriptome assembly for each group (Table S2). A total of 585 871 genes encoding 783 913 transcripts, 384 463 genes encoding 523 710 transcripts, and 941 343 genes encoding 1 203 188 transcripts were assembled using Trinity [44] for Groups 1, 2, and 3, respectively. Multiple filtering steps were carefully performed to obtain reliable new genes (Figure S1 and Table S2). For each gene, only the longest transcript sequence was retained as a representative sequence of this gene for further filtering. After filtering short sequences (<500 bp), redundant loci, and low-expression genes, 9910, 9894, and 5797 genes were retained in Groups 1, 2, and 3, respectively. All genes in the three groups were merged to further remove redundant sequences between groups, and 9130, 9061, and 5124 genes were retained. A total of 405, 355, and 192 genes were defined as potential contaminant sequences because they showed significant similarity to bacterial, fungal, and viral sequences and were removed. Finally, a total of 4940 genes (1952 from Group 1, 1471 from Group 2, and 1517 from Group 3) were retained after removal of sequences with low coding potential (<100 aa) and putative alleles of TGY genes (Table S2).

### Population-level expression variations of the pan-transcriptome

**Figure 2:**
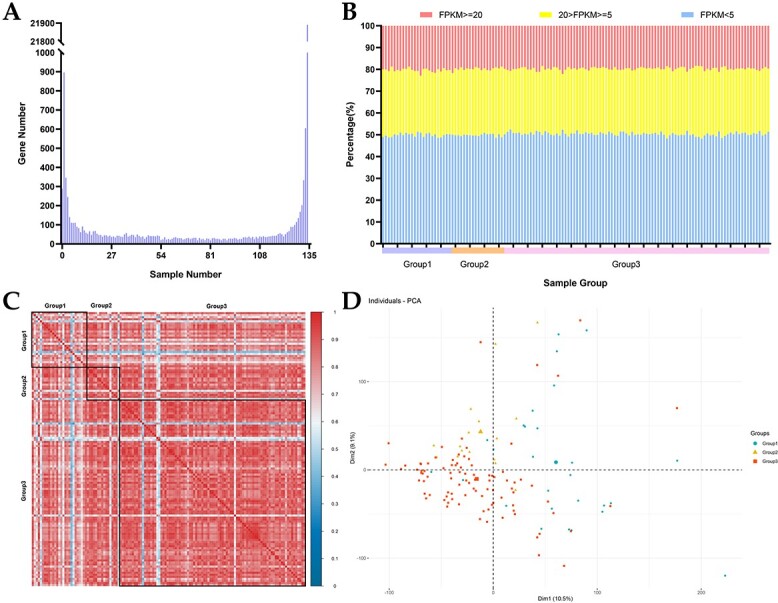
Variations in gene expression among the three populations. A. Expression distribution of genes among 134 tea plant accessions. B. Proportion of genes expressed at different levels. C. Pearson correlations of 134 tea plant accessions based on the expression levels of all genes. D. Principal component results (first (Dim1) and second (Dim2) principal components) of 134 tea plant accessions based on the expression levels of all genes.

To analyze expression variation at the tea plant population level, we analyzed the expression distribution and expression abundance of all genes in the pan-transcriptome. A total of 21 891 genes (defined as core expressed genes) were expressed (FKPM >0) in 134 accessions, 23 549 genes were expressed in more than 95% of accessions (>127 accessions), 896 genes were expressed in only one accession (accession-specific genes), and 1959 genes were expressed in fewer than 7 accessions (approximately 5% of the total accessions) (Figure 2A). Core expressed genes and accession-specific genes were enriched in different KEGG pathways, core expressed genes biased toward pathways for essential cellular functions. Accession-specific genes were also related to specific biological pathways such as fructose and mannose metabolism, glutathione metabolism, signaling, and cellular processes (Figure S3).

We next classified genes with FPKM ≥ 20, 20 > FPKM ≥ 5, and 5 > FPKM as highly expressed (HEGs), medium-expressed (MEGs), and low-expressed (LEGs) genes, respectively. The proportions of HEGs, MEGs, and LEGs were relatively similar among all accessions, and in general, the proportion of LEGs was much higher than that of HEGs and MEGs (Figure 2B). Here, 1865 HEGs, 500 MEGs, and 6138 LEGs were shared among all 134 accessions. In total, 38 KEGG terms (31 + 7) enriched in the HEGs and MEGs belonged to four main classes: metabolism, genetic information processing, cellular processes, and brite hierarchies, which are indispensable biological processes of plants (Table S3). By contrast, LEGs were significantly enriched in the main class of organic systems, including plant pathway interactions and environmental adaptation, suggesting that LEGs play an important role in plant adaptation to biotic or abiotic stresses (Table S3) and that their expression may be induced by biotic or abiotic stresses and maintained at a low expression level in normal environments.

The results of the correlation and PCA of all gene expression levels did not clearly distinguish Groups 1, 2, and 3, owing to the existence of non-pure CSA or CSS accessions in Groups 1 and 2 or to many genes that were not expressed in a group-specific manner among these tea plants (Figure 2C and 2D). We therefore analyzed the differentially expressed genes (DEGs) between pure CSA and CSS accessions (Figure S4A) and found that these DEGs could clearly distinguish Groups 1, 2, and 3 (Figure S4B), as well as pure CSA and pure CSS (Figure S4C). This result indicated that these DEGs may play important roles in the phylogenetic differentiation process of tea plants. We identified 1378 and 1060 upregulated DEGs (Up-DEGs) in pure CSA and pure CSS, respectively (Figure S4A), and these DEGs were enriched (P < 0.05) in different GO terms and pathways (Figure S4D and S4E). Up-DEGs in pure CSA had a higher number of GO terms (43) than Up-DEGs in pure CSS (Figure S4D). Interestingly, Up-DEGs in pure CSS were significantly enriched in eight GO terms, and these GO terms were all closely related to specialized metabolites, implying that they may play essential roles in the metabolism of CSS specialized metabolites (Figure S4D). The KEGG enrichment analysis also showed that Up-DEGs in pure CSA involved a greater number of biological pathways than Up-DEGs in pure CSS, suggesting broad-spectrum effects for tea plants, whereas Up-DEGs in pure CSS were specifically enriched in pathways related to specialized metabolite synthesis, including ABC transporters, carotenoid biosynthesis, glycosyltransferases, pentose and glucuronate interconversions, sesquiterpenoid and triterpenoid biosynthesis, and zeatin biosynthesis (Figure S4E).

PAVs and CNVs make a great contribution to plant phenotypic diversity and determinism [25]. We therefore analyzed the genes specifically expressed in pure CSA or CSS accessions and defined them as CSA-ePAVs (expressed only in CSA) or CSS-ePAVs (expressed only in CSS), which resulted in 210 and 154 CSA-ePAVs and CSS-ePAVs (Figure S4A). The CSA-ePAVs were enriched in genes mainly related to the response to acid chemicals (Figure S4F), whereas CSS-ePAVs were significantly enriched in protein phosphorylation, phosphorylation, phosphate-containing compound metabolic processes, and phosphorus metabolic processes (Figure S4G). These results indicated that CSA and CSS have clearly differentiated at the population transcriptome level, and this differentiation was closely related to the characteristic phenotypes of CSA and CSS.

### Specialized metabolite differences between pure CSA and pure CSS

**Figure 3:**
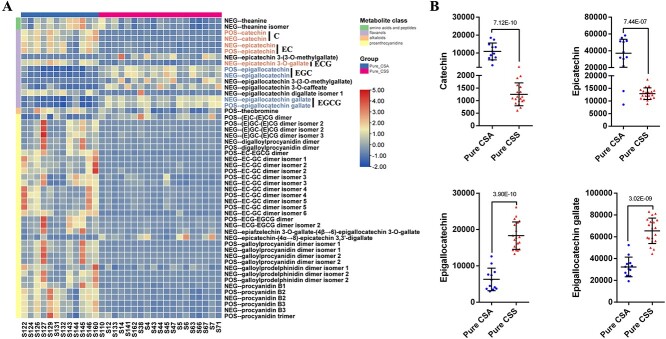
Metabolite variations between pure CSA and pure CSS. A. Differentially accumulated signatures (DASs) of amino acids and peptides, flavanols, alkaloids, and proanthocyanidins between pure CSA and pure CSS. B. Scatter dot plots of the content distribution of catechin, epicatechin, epigallocatechin, and epigallocatechin gallate in POS mode between pure CSA and pure CSS (Student’s t-test).

Differences in specialized metabolites between tea plant leaves from CSA and CSS determine their suitability for making different types of processed tea and the different flavors of the resulting tea products [1]. A total of 117 specialized metabolite signatures were identified as differentially accumulated signatures (DASs) between pure CSS and pure CSA (present in >8 samples, fold change > 2 or < 0.5, P-value < 0.01) involving 82 specialized metabolites (Table S4). Several previously uncharacterized DASs between CSA and CSS were discovered. The content of theanine and theobromine in pure CSA was higher than that in pure CSS, and most specialized metabolites belonging to the proanthocyanidins also had a higher content in pure CSA than in pure CSS (Figure 3A). Specialized metabolites belonging to the flavanols showed different but interesting distribution patterns. Catechin (C) and epicatechin (EC) had higher levels in CSA than in CSS, whereas epigallocatechin (EGC) and epigallocatechin gallate (EGCG) had higher levels in CSS (Figure 3A and 3B). In addition, several CSS- or CSA-specific specialized metabolites were highlighted, including dihydrocaffeic acid 3-O-glucuronide, di-p-cis-coumaroylquinic acid isomer 1, di-p-cis-coumaroylquinic acid isomer 2, quercetin 3-O-galactosyl rutinoside, epigallocatechin 3-(3-O-methylgallate), and methyl galloylglucose in pure CSS and quercetin hexose deoxyhexose and galloylprodelphinidin dimer isomer 2 in pure CSA (Table S4).

### Candidate genes and regulatory network of specialized metabolites in tea plants

**Figure 4:**
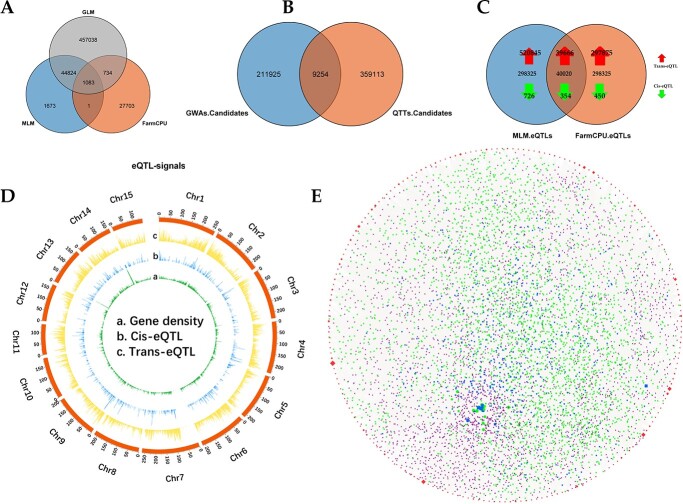
The regulatory network of tea plant specialized metabolites. A. Venn analysis of GWAS signals of tea plant specialized metabolites from GLM, MLM, and FarmCPU methods. B. Venn analysis of candidate genes from QTTS and GWAS. C. Venn diagram of eQTLs from the MLM and FarmCPU methods. D. Genomic distribution of gene density (a), Cis-eQTLs (b), and Trans-eQTLs (c) of all genes in the tea plant pan-transcriptome. E. The regulatory network of tea plant specialized metabolites, consisting of 212 metabolites (the red dots in the outermost circle), 3283 candidate genes (green dots inside the circle), 2847 eQTLs (purple dots inside the circle), and 560 that are both candidate genes and eQTLs (blue dots inside the circle).

A total of 248 specialized metabolites related to 342 annotated metabolic features from the POS and NEG modes of these 134 tea plant accessions were downloaded from the EMBL European Bioinformatics Institute (EBI) (project number: MTBLS1405) [1] and used for a metabolome-based genome-wide association study (mGWAS) (Table S5). A total of 46 642 significant SNPs associated with these specialized metabolites were colocalized by more than two GWAS models (Figure 4A). In addition, a quantitative trait transcript study (QTTS) was used to associate genes with the specialized metabolites, and the results revealed 368 367 gene–metabolite pairs (Figure 4B).

To obtain highly credible candidate genes for specialized metabolites, we constructed Venn diagrams from the results of GWAS and QTTS. Only genes that were within 1.5 Mb upstream and downstream of significant SNPs (a total of 221 179 genes) and simultaneously colocalized by QTTS were selected as the final specialized metabolite candidate genes, resulting in 9254 gene–metabolite pairs (3843 genes associated with 212 specialized metabolites, Figure 4B**,**  Table S6). Among the 3843 genes, 78 candidate genes were associated with more than ten specialized metabolites, suggesting that they play important roles in the tea plant specialized metabolite network (Table S7).

To identify eQTLs of genes at the pan-transcriptome level, all genes in the pan-transcriptome were used as traits for GWAS analysis. A total of 40 020 eQTLs were co-identified from the MLM and FarmCPU methods at the pan-transcriptome scale, including 39 666 Trans-eQTLs and 354 Cis-eQTLs (Figure 4C). The Trans-eQTLs and Cis-eQTLs were not evenly distributed on the 15 chromosomes (Figure 4D). A total of 1580 of the 3843 candidate genes associated with 212 specialized metabolites had 3407 eQTLs containing 12 047 candidate gene–eQTL links (Table S8). We first constructed a specialized metabolite regulatory network of tea plants consisting of 212 metabolites, 3843 candidate genes, and 3407 eQTLs (Figure 4E); 560 genes were both candidate genes and eQTLs (Figure S5). In this network, we defined eQTLs associated with more than 10 candidate genes as candidate gene-rich eQTLs and identified 303 candidate gene-rich eQTLs, accounting for 8.9% of the total eQTLs (Table S9). The annotation results revealed that most of these candidate gene-rich eQTLs encoded enzymes related to the synthesis of specialized metabolites. These candidate gene-rich eQTLs also contained 18 transcription factors (TFs), including four WD40, two MYB-related, one bHLH, and one WRKY TF (Table S9). These TFs have previously been characterized to regulate a variety of specialized metabolites [45, 46]. We further identified 382 TFs in this regulatory network (Figure S6). The top 5 TFs based on total number were members of the AP2/ERF-ERF (25), MYB (23), WD40 (22), bHLH (20), and C3H (19) families, suggesting that these transcription factors play important roles in the regulation of tea plant specialized metabolites.

### *CsTGY14G0001296* is an important candidate gene for proanthocyanin variation in tea plants

**Figure 5:**
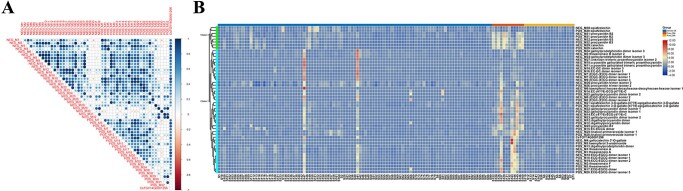
The Pearson correlation coefficients (A) and heatmap (B) of *CsTGY14G0001296* with 32 tea plant specialized metabolites obtained by colocalization of QTTS and GWAS.

We mentioned earlier that most proanthocyanins were higher in CSA than in CSS (Figure 3A). However, the molecular basis for this difference is unclear. Here, the GWAS and QTTS colocalized to a gene (*CsTGY14G0001296*) associated with 32 specialized metabolites, including 23 proanthocyanins, three bisflavanols, three flavanols, two flavonol glycosides, and one terpenoid glycoside (Table S10). The Pearson correlation coefficients between *CsTGY14G0001296* and these 32 specialized metabolites revealed that *CsTGY14G0001296* was significantly positively correlated with all of the specialized metabolites except for catechin, procyanidin B2, procyanidin B3, and epiafzelechin (Figure 5A). The heatmap also showed that *CsTGY14G0001296* and these 28 specialized metabolites clustered into Cluster 1, whereas catechin, procyanidin B2, procyanidin B3, and epiafzelechin clustered into Cluster 2 (Figure 5B).

We further analyzed the function of *CsTGY14G0001296* based on its KEGG annotation and found that it was an ortholog of *Arabidopsis AtANS* (AT4G22880), which encodes leucoanthocyanidin dioxygenase and is associated with proanthocyanin biosynthesis [47]. Similar to our expression results, Zhang et al. (2019) previously found that the expression level of *AtANS* was positively correlated with proanthocyanin content, and *AtANS*-deficient *Arabidopsis* mutants had lower proanthocyanin contents than wild-type plants [47]. In cacao, overexpression of *TcANS* in tobacco resulted in increased contents of both anthocyanidins and proanthocyanins in their flower petals, and overexpression of *TcANS* in an *Arabidopsis ldox* mutant complemented its proanthocyanin-deficient phenotype in seeds [47]. Our results from the association and expression analysis provided multidimensional evidence that *CsANS* in tea plants may have a similar function in the regulation of proanthocyanins, and this *ANS*-related mechanism is likely to be conserved among multiple plant species. This implies that the content of proanthocyanins in tea plants can be regulated by manipulating the expression level of the *CsANS* gene. Interestingly, a previous report on heterologous transgenic insertion of *CsANS* into tobacco supports our inference. As expected, *CsANS*-overexpressing tobacco plants produced more proanthocyanins than wild-type plants [48]. The transgenic system of tea plants is at an early stage of development, and this speculation will require further verification using *CsANS* transgenic tea plants in the future.

Excessive accumulation of anthocyanins can cause plant tissues to appear purple [47]. We found that the accessions with excessive accumulation of proanthocyanins, such as S147 and S41 “Zi juan” and S127 “Zi Ya”, also had purple leaves. Similarly, previous studies have demonstrated that the expression level of *CsTGY14G0001296* is higher in purple-leaf cultivars than in green cultivars [49–51]. In carnations, the *DcaANS* gene also showed higher expression in pink/red-flowered cultivars than in white-flowered cultivars [52]. In lisianthus, a 94-bp deletion in the *ANS* gene led to white-flowered lines [53]. In addition, *LsANS*-overexpressing *Arabidopsis* plants had a red color in multiple organs [54]. In summary, *CsTGY14G0001296* is the *ANS* gene in tea plants and can explain differences in proanthocyanin content and purple leaf color among tea populations. These findings will broaden our understanding of the functions of *ANS* in tea plants.

### Genetic architecture of flavanol variation in tea plants

Various types of catechins and theasinensins are flavanols that are responsible for the unique flavors of tea [5, 9]. To dissect the biased distribution of C, EG, ECG, EGC, and EGCG (Figure 3A and 3B) between pure CSA and pure CSS, we integrated the pan-transcriptome DEG results, GWAS QTL results, and QTTS QTL results.

**Figure 6:**
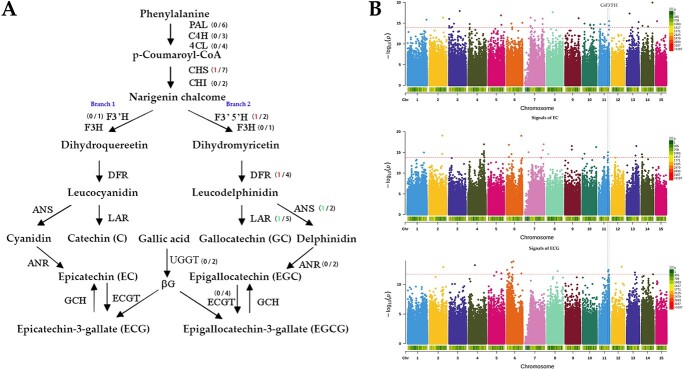
An important regulatory factor for flavanol variation. A. Differentially expressed structural genes in the flavonoid biosynthesis pathway. The first number in parentheses represents differentially expressed genes (upregulated in red and downregulated in green), and the second number is the total number of each gene. B. *F3’5’H* is a candidate gene for catechin, epicatechin, and epicatechin-3-gallate from a genome-wide association study (GWAS). The horizontal red line indicates the significance threshold from 100 repetitions of the permutation test in rMVP.

A total of 53 flavonoid biosynthesis pathway structural genes were identified in the pan-transcriptome (Figure 6A and Table S11). Five of these genes were differentially expressed (|log2FoldChange| > 2 and FDR < 0.01) in pure CSS and pure CSA. *CHS*, *F3′5′H* (*CsTGY11G0002074*), and *DFR* had a higher expression level in pure CSS than in pure CSA, whereas *LAR* and *ANS* had a higher expression level in pure CSA than in pure CSS. Because *CHS*, *DFR*, *LAR*, and *ANS* are not at divergent nodes, their expression changes have the same impact on branch 1 and branch 2, which does not lead to a two-level deviation of the products of the two branches. *F3′5′H* and *F3′H* are the initial enzymes of branch 1 and branch 2, and they have a competitive relationship. *F3′5′H* is very likely to be the main factor leading to the biased distribution of C, EG, ECG, EGC, and EGCG between pure CSA and pure CSS. This speculation was further confirmed by the results of GWAS QTLs and QTTS QTLs: *F3′5′H* was a candidate gene for GWAS QTLs of C, EG, and ECG and a candidate gene for QTTS QTLs of EGCG (Figure 6B). In summary, the higher expression of *F3′5′H* in pure CSS led to more products of branch 2 (EGC and EGCG). Conversely, pure CSA produced more products of branch 1 (C, EC, and ECG). We noticed that there were 6 SNPs in the region 2 kb upstream of the *CsTGY03G0000951* gene, 4 of which had an Fst value of 1 (pure CSA vs. pure CSS) (Table S12), implying that the upstream regulatory region of *CsTGY03G0000951* has completely differentiated between CSA and CSS.

One gene (*CsTGY03G0000951*) on chromosome 3 encoding an NAC TF was identified as an eQTL of *CsTGY11G0002074*, suggesting that it may regulate the expression of *CsTGY11G0002074* (Figure S7). In addition to *CsTGY11G0002074*, multiple TFs were identified as candidate genes for C and EC, including two AP2/ERF-ERF (*CsTGY11G0001570* and *CsTGY12G0001883*) TFs for C and one MYB-related TF (*CsTGY14G0001296*) and one TCP TF (*CsTGY15G0001531*) for EC (Table S13). These potential regulatory factors provide reliable targets for future functional verification.

## Discussion

mGWAS has been used to study the genetic variation of high-throughput specialized metabolites in maize [18, 19], rice [20, 21], foxtail millet [55], wheat [56, 57], and other species [58]. However, apart from a few well-known specialized metabolites, namely catechins, caffeine, and theanine [9, 10], other specialized metabolites in tea plants have rarely been studied. To our knowledge, a comprehensive association analysis of genotypes, metabolites, and gene expression levels has not been performed previously [1]. In the current research, we integrated multi-omics data to understand the genetic basis, important candidate genes, and regulatory network of specialized metabolite differences [1]. Because QTL mapping and GWAS analysis are not sufficient to explain all phenotypic variations [25, 26] and because phenotypic variation is also closely related to DEGs, ePAVs, and CNVs [27–29], we assembled the first pan-transcriptome of tea plant leaves and defined several pure CSA and pure CSS based on the evolutionary tree and the population structure. Comparative transcriptome results based on the pan-transcriptome showed that DEGs and ePAVs between CSA and CSS played an important role in the variation of tea plant traits.

Owing to the inevitable limitations of methods that involve a single data type, the integration of multi-dimensional datasets such as genotype, metabolite, and gene expression data can compensate for missing or unreliable information in any single data type [59]. Multi-dimensional analysis is regarded as an important means of exploring biological mechanisms and can provide systematic clues for understanding complex biological problems [57, 60, 61]. For instance, Zhu et al. (2017) analyzed genotype, transcriptome, and metabolome datasets from 610 tomato accessions, highlighting the impact of global breeding on tomato metabolite content [22]. Here, we used multi-dimensional analysis of genomes, pan-transcriptomes, and metabolomes to construct a global specialized metabolite regulatory network and highlighted several important genes that regulate multiple specialized metabolites. We provided more evidence for the role of *CsTGY14G0001296* in determining differences in the accumulation of proanthocyanidins in pure CSA and pure CSS. In addition, many candidate gene-rich eQTLs were identified, and some candidate gene-rich eQTLs were consistent with previous studies [5], encoding enzymes related to specialized metabolite pathways, such as tripeptidyl-peptidase, cinnamoyl-CoA reductase, and E3 ubiquitin-protein ligase (Table S8). Important candidate gene-rich TFs in the regulatory network of tea plant specialized metabolites were highlighted by the multi-omics analysis, includingAP2/ERF-ERF, MYB, WD40, bHLH, and C3H TFs. Some have previously been characterized by heterologous or homologous transgenic experiments [62].

The MYB-bHLH-WD40 ternary complex is involved in the synthesis of plant polyphenols, catechins, and proanthocyanidins [2, 63–66]. At least three MYB TFs have been experimentally characterized in relation to polyphenol biosynthesis: CsMYB4a negatively regulates the phenylpropanoid and shikimate pathways [67], CsMYB5 regulates anthocyanin and proanthocyanidin biosynthesis [45], and CsMYB75 regulates anthocyanin hyperaccumulation [46]. The global regulatory network of tea plant specialized metabolites revealed that the number of TFs related to specialized metabolites is considerable, far exceeding the current single-digit number. It includes some TFs that have not been verified in tea plants, including AP2/ERF-ERF and C3H TFs. The results of DEG and association analyses all indicated that *F3′5′H* (*CsTGY11G0002074*) was associated with the biased distribution of C, EG, ECG, EGC, and EGCG between pure CSA and pure CSS. Analysis of the Fst values of SNPs upstream of this gene showed that the upstream promoter regions were quite different between CSA and CSS and may explain the difference in expression. Recently [68], a novel *F3′5′H* allele with a 14-bp deletion was reported to be associated with a high catechin index trait in wild tea plants, and the 14-bp deletion in the novel *F3′5′H* allele was associated with low *F3′5′H* mRNA expression [68]. These results suggest that *F3′5′H* can be used as a target for manipulating the types and contents of catechins. We also showed that an NAC TF (CsTGY03G0000951) may be one of the upstream regulatory genes of *F3′5′H*. Other TFs related to catechins were also revealed: two AP2/ERF-ERF (CsTGY11G0001570 and CsTGY12G0001883) TFs for C and one MYB-related TF (CsTGY14G0001296) and one TCP TF (CsTGY15G0001531) for EC. Together, our multi-omics results lay a foundation for understanding the genetic mechanism of specialized metabolites, and the newly identified genes provide alternative targets for future tea quality improvement.

The transcriptome is one mediator between the genome and the phenotype, and it is an ideal tool for constructing a gene network to analyze phenotypic differences in the tea population. However, earlier studies on tea plant transcriptomics remain at the level of a single reference genome. Comparative transcriptomics analysis based on a single reference genome will be missing important information about the differentiation of traits because a single reference genome represents only a part of the species genome [23, 25, 26, 34]. Therefore, it is useful to carry out population-level comparative transcriptome analysis based on the pan-genome or pan-transcriptome. The first pan-transcriptome of tea leaves assembled here provides usable reference sequences for future population-level comparative transcriptomics studies. Because of the self-incompatibility characteristics of tea plants, excellent tea varieties are bred mostly through intra- and inter-specific crosses [43], resulting in a small number of pure CSA and CSS. Based on the evolutionary tree and population structure, we obtained several pure CSA and CSS accessions and identified DEGs and ePAVs between pure CSA and CSS. Interestingly, the DEGs and ePAVs of CSS and CSA were significantly enriched in different pathways, consistent with their growth characteristics. This finding indicates that these biased genes are related to the differentiation of CSA and CSS.

Through the cleverly designed hybridization of CSA and CSS to aggregate these expression-biased genes, it is possible to breed elite tea varieties with excellent flavor and high tolerance to stresses. Notably, we found that LEGs in the pan-transcriptome were significantly enriched in plant pathway interactions and environmental adaptation, which can be an excellent mechanism for energy savings and self-protection of tea plants. Genes related to environmental adaptation are induced by a variety of stresses, and low expression levels under ideal conditions can help reduce energy consumption [69]. The accumulation of stress resistance metabolites caused by expression of environmental adaptation-related genes hinders the normal growth of plants, and these genes therefore tend to be induced by stresses [70].

In conclusion, this study provides important insights into the genetic variation underlying the differences in specialized metabolites among tea plants and offers further directions for crop breeding of tea plants. Our newly developed pan-transcriptome resources can advance molecular biology research, and the identified candidate genes for specialized metabolites can be used to improve the quality of tea and the stress tolerance of tea plants in the immediate future.

## Materials and methods

### Transcriptome and metabolomics data

The RNA-seq data for fully expanded second leaves from young shoots (one bud with two leaves) of 136 representative tea accessions (from 128 cultivars) were generated by a previous study [1] and were downloaded from the National Center for Biotechnology Information (NCBI, project number PRJNA562973). Metabolomics data for these 136 representative tea accessions were obtained from the EMBL-European Bioinformatics Institute (EBI, project number MTBLS1405) [1].

### SNP calling, clustering analysis, and population structure

Raw RNA-seq data were filtered using fastp (https://github.com/OpenGene/fastp) with default parameters. Quality-controlled reads were mapped to the monoploid TGY genome (https://ngdc.cncb.ac.cn/search/?dbId=gwh&q=GWHASIV00000000) using HISAT2 software [71]. PCR duplicate reads were then filtered with the MarkDuplicates function in Picard, and retained reads were used to call variants with default parameters of the HaplotypeCaller, CombineGVCFs, and GenotypeGVCFs functions built into GATK 4.2.0.0 [72]. Next, SNPs were extracted and filtered using the SelectVariants function with default parameters and the VariantFiltration function with the parameters “QD < 2.0 || MQ < 40.0 || FS > 60.0 || SOR > 3.0 || MQRankSum < -12.5 || ReadPosRankSum < -8.0” in GATK 4.2.0.0. Finally, all SNPs were further filtered using PLINK v1.9 with parameters “maf 0.05, geno 0.3”, and 2 285 251 chromosome-anchored SNPs were retained. All SNPs were annotated using snpEff (https://sourceforge.net/projects/snpeff/files/).

The 2 285 251 chromosome-anchored SNPs were filtered to 643 296 SNPs according to SNP linkage relationships using PLINK v1.9 with the parameter “indep-pairwise 50 10 0.2” for phylogenetic tree construction and population structure analysis. A maximum likelihood-based phylogenetic tree was built from 643 296 SNPs using IQ-TREE (http://www.iqtree.org/) with the GTR model and 1000 bootstrap replications [73]. The population structure of tea plants was inferred with ADMIXTURE software using K = 2–5 [74].

### Pan-transcriptome construction

A detailed schematic diagram of pan-transcriptome construction is provided in Figure S1. The reads successfully mapped by HISAT2 software were assembled with StringTie-1.3.3b [75] for each accession. The transcripts assembled from each accession were combined to obtain the transcripts for each group using taco-v0.7.3 [76]. The three sets of group-level transcripts were merged into tea plant population-level transcripts (primary pan-transcriptome) with StringTie-1.3.3b [75]. The expression levels of all transcripts were calculated as FPKM using Ballgown [71], and transcripts with an average FPKM (total FPKM/total samples) less than 1 were filtered to form a filtered pan-transcriptome for requantification of all genes.

The unmapped reads from each group were extracted with SAMtools [77] for *de novo* assembly. Unmapped reads from each group were assembled using Trinity v2.9.1 [44] with a minimum assembled contig length of >500 bp. To remove redundant transcripts for each locus, the longest transcript within a locus was defined as the representative transcript [25, 26]. Then, redundant sequences of representative transcripts were removed using CD-HIT-EST (https://github.com/weizhongli/cdhit/releases) with parameters “-c 0.95 -n 10”. Nonredundant sequences were used for re-quantitative analysis of reads using align_and_estimate_abundance.pl in Trinity v2.9.1 with the parameters “--est_method RSEM --aln_method bowtie”, and loci with low expression levels (FPKM/per sample < 20) were deleted.

Next, the remaining loci in the three groups were merged to remove redundant sequences using CD-HIT-EST [78] and potential contaminant sequences by searching against a total of 3 036 338 bacterial, fungal, and viral sequences downloaded from NCBI (blastn, -evalue 1e-5 -perc_identity 50.00) (https://www.ncbi.nlm.nih.gov/). The open reading frames (ORFs) of the remaining sequences were predicted with TransDecoder (http://transdecoder.github.io/), and those that encoded protein sequences less than 100 amino acids in length were removed. Finally, putative allele sequences with TGY sequences were filtered based on blastn alignment with a similarity of >80% [25].

### Identification and annotation of DEGs and ePAVs

DEGs between pure CSS and pure CSA were identified using DESeq2 with p-value < 0.01 and |log_2_(fold change)| ≥ 2. Genes expressed only in pure CSS or pure CSA were defined as ePAVs. EggNOG-mapper (http://eggnog5.embl.de/#/app/home) was used to annotate all genes in the pan-transcriptome, and Taxonomic Scope was set to Viridiplantae [79]. Gene function enrichment of DEGs and ePAVs was performed by GO and KEGG enrichment analysis in TBtools v1.098661 [80].

### GWAS and QTTS of specialized metabolites

Genotype imputation of 2 285 251 chromosome-anchored SNPs was performed with Beagle 5.2 [81]. The genome-wide LD decay of all SNPs was estimated with PopLDdecay [82]. Two univariate GWAS methods (GLM and MLM) and one multivariate GWAS method (FarmCPU) were used to evaluate the trait–SNP associations for 342 annotated specialized metabolites using rMVP with the parameters “K=Kinship, nPC. GLM=5, nPC. MLM=3, nPC. FarmCPU=3, priority=speed, vc.method=BRENT, maxLoop=10, method.bin=static, threshold=0.05” [83].

We used a linear regression model (lm() function in R) to identify QTTS correlated with specialized metabolites based on the method described by Pang et al. (2019) [84]. The first three principal components of SNPs were added to the linear regression model as a covariate [84]. We used a P-value <0.01 as the cut-off for the QTTS in this study.

Finally, genes that were 1.5 Mb upstream and downstream of significant SNPs and colocalized by QTTS were selected as the candidate genes for specialized metabolites in the tea plant specialized metabolite regulation network.

### eQTL identification of TWAs

The expression levels of all genes in the pan-transcriptome were regarded as traits for GWAS to identify eQTLs using the MLM and FarmCPU methods. Genes linked by QTN were recorded as Cis-eQTLs if their physical distance was less than 100 kb from the genes used as traits and as Trans-eQTLs if this distance exceeded 100 kb.

## Supplementary Material

Web_Material_uhac100

## Data Availability

All the original data for this study were provided by Prof. Liu [1], and these data are publicly available at the National Center for Biotechnology Information (NCBI) with project number PRJNA562973 or the EMBL-European Bioinformatics Institute (EBI) with project number MTBLS1405.
