# Supplementary material for: Pan-transcriptome assembly combined with multiple association analysis provides new insights into the regulatory network of specialized metabolites in the tea plant Camellia sinensis
Source: Hortic Res. 2022 Jul 2;9:uhac100. doi: 10.1093/hr/uhac100 (PMC9251601; doi:10.1093/hr/uhac100)
Supplement: Web_Material_uhac100 [file web_material_uhac100.zip › Editing Certificate.pdf]

Editing Certificate

This document certifies that the manuscript

Pan-transcriptome assembly combined with multiple association analysis provides new insights into the regulatory network of specialized metabolites in tea plant, Camellia sinensis

prepared by the authors

Kong Weilong

was edited for proper English language, grammar, punctuation, spelling, and overall style by one or more of the highly qualified native English speaking editors at AJE.

This certificate was issued on **April 11, 2022** and may be verified on the AJE website using the verification code **1FBC-42E3-14C6-D2F9-1983**.

Neither the research content nor the authors' intentions were altered in any way during the editing process. Documents receiving this certification should be English-ready for publication; however, the author has the ability to accept or reject our suggestions and changes. To verify the final AJE edited version, please visit our verification page at aje.com/certificate. If you have any questions or concerns about this edited document, please contact AJE at support@aje.com.

AJE provides a range of editing, translation, and manuscript services for researchers and publishers around the world. For more information about our company, services, and partner discounts, please visit aje.com.
