# Supplementary material for: Pan-transcriptome assembly combined with multiple association analysis provides new insights into the regulatory network of specialized metabolites in the tea plant Camellia sinensis
Source: Hortic Res. 2022 Jul 2;9:uhac100. doi: 10.1093/hr/uhac100 (PMC9251601; doi:10.1093/hr/uhac100)
Supplement: Web_Material_uhac100 [file web_material_uhac100.zip › Figure S.docx]

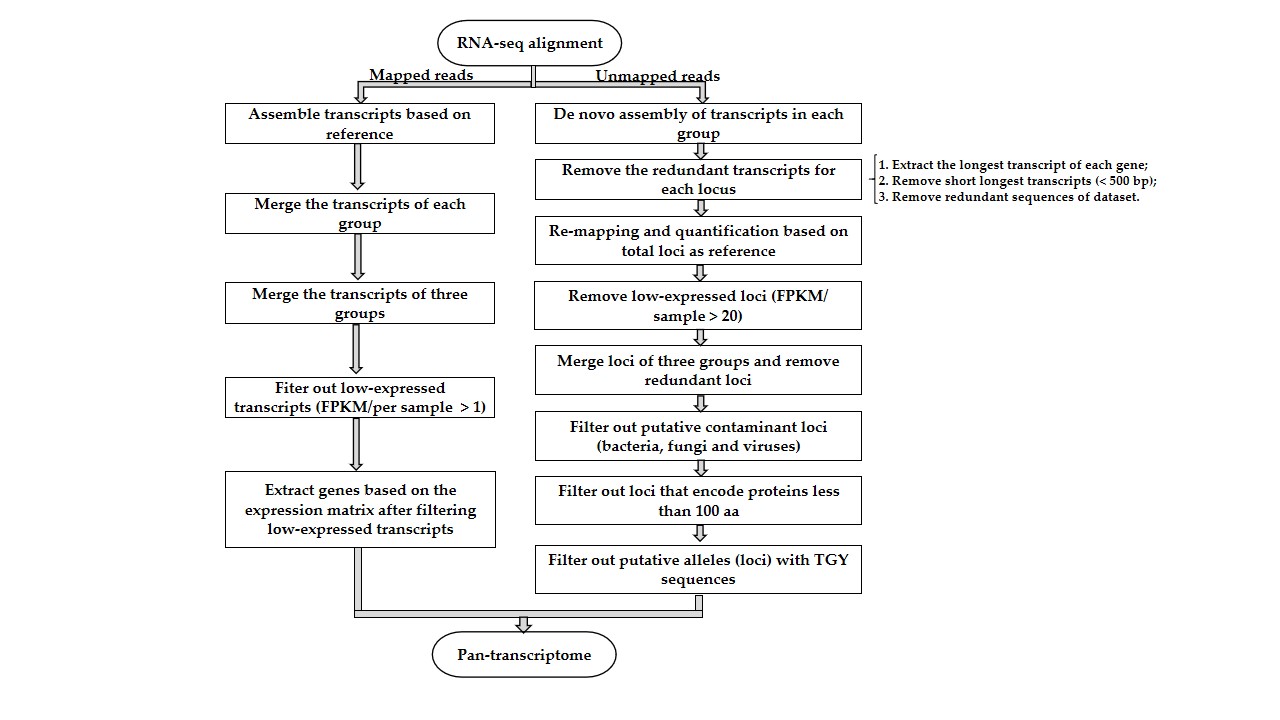


**Figure S1. Pipeline for the pan-transcriptome assembly**

**
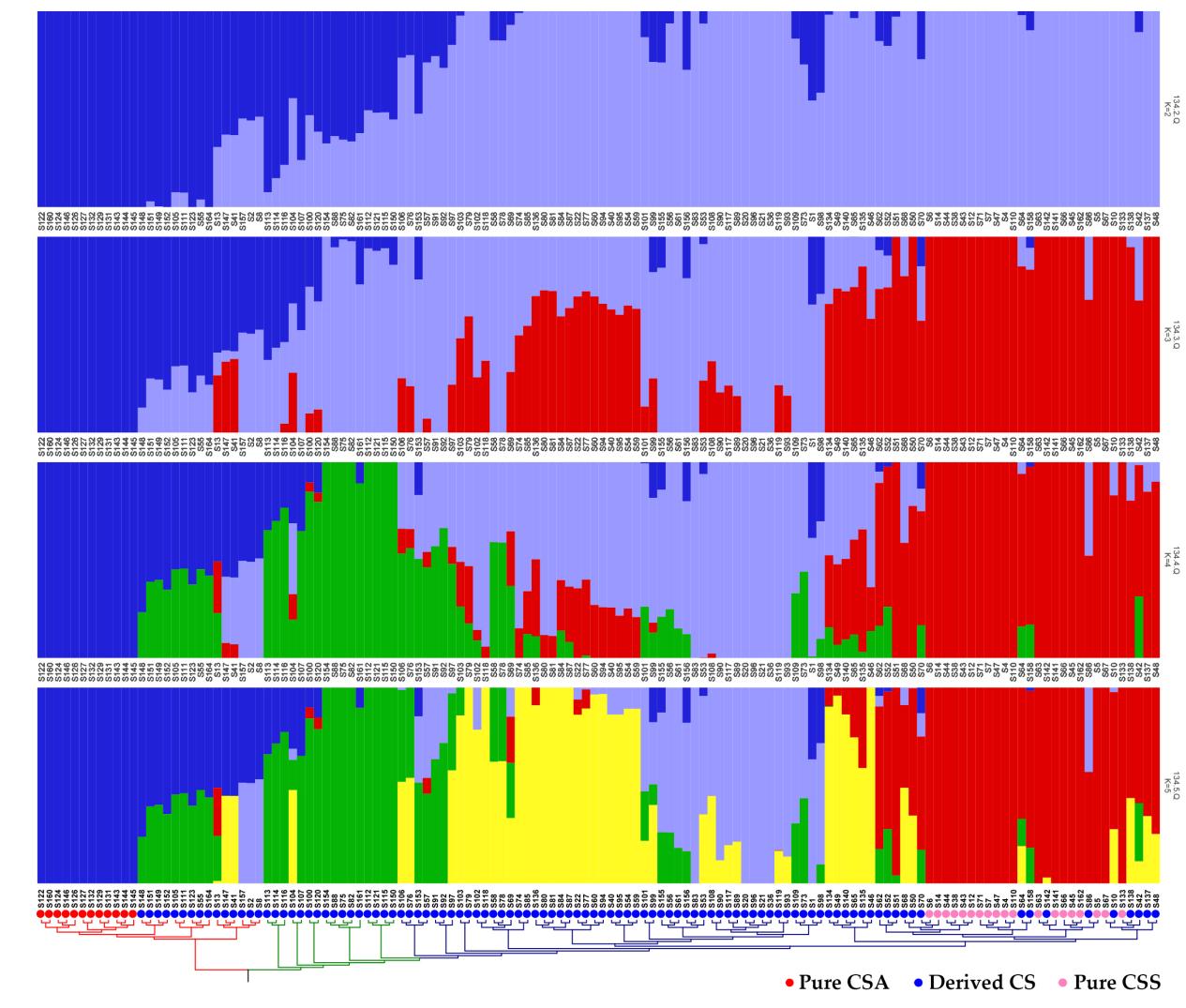
**

**Figure S2. Group structure result of K = 2 – 5.** **Different colors represent genetic backgrounds of different subpopulation.**


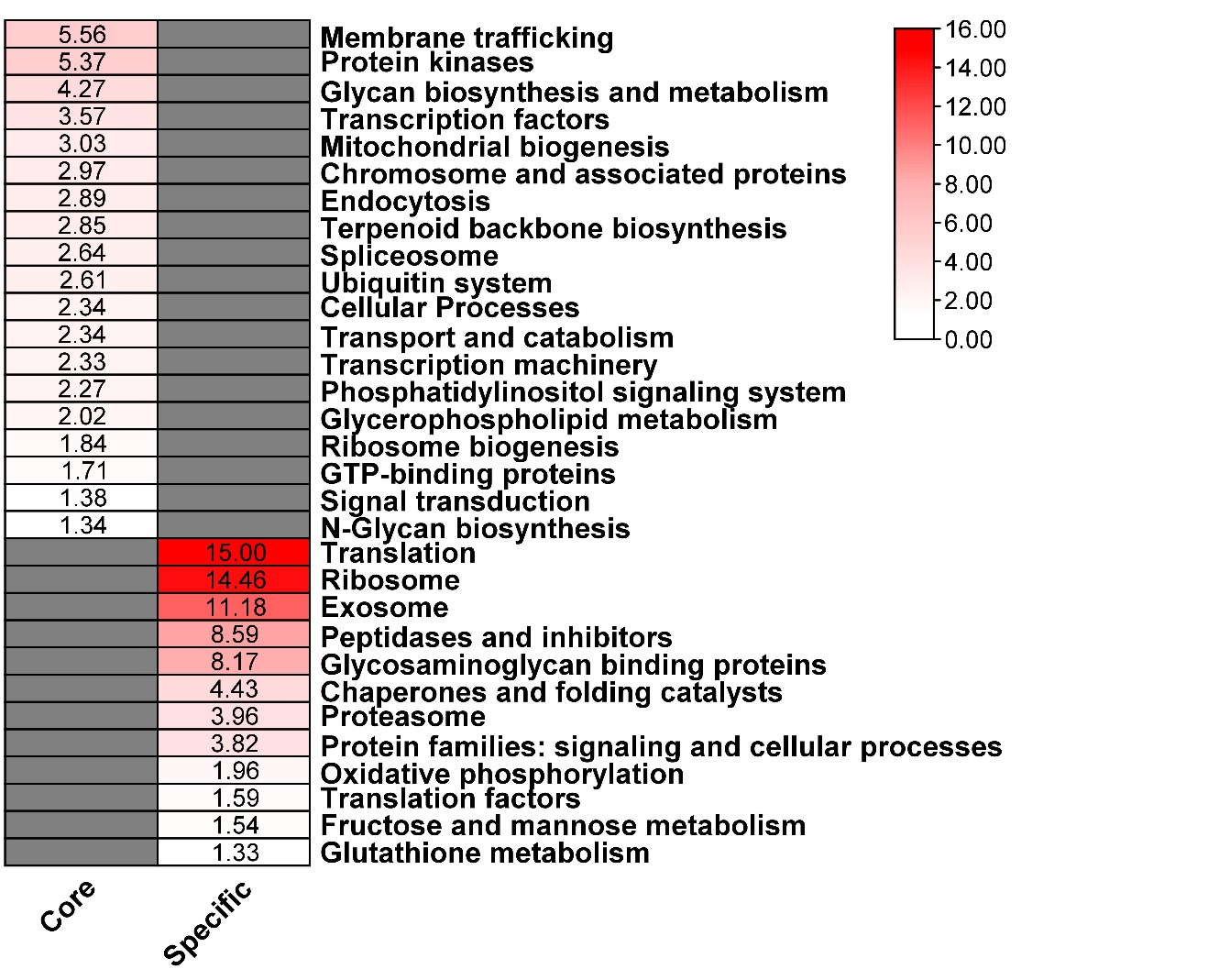
**Figure S3. KEGG enrichment of core expressed genes and cultivar specific genes in pan-transcriptome of tea plant leaf**


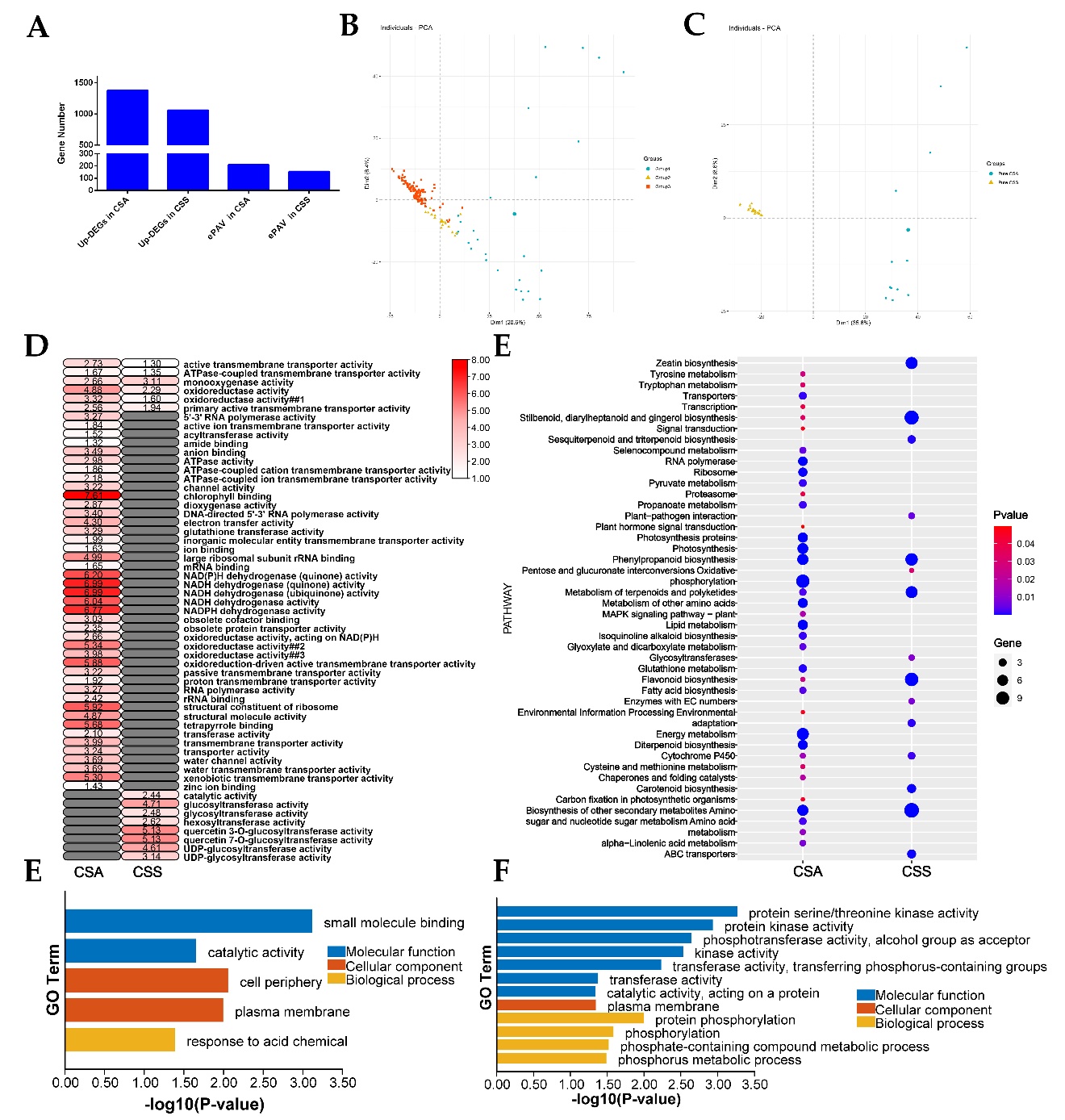


**Figure S4. The differentially expressed genes (DEGs) and expression presence/absence variations (ePAVs) between pure *C. sinensis* var. *assamica* (CSA) and pure *C. sinensis* var. *sinensis* (CSS). A.** The numbers of DEGs and ePAVs between pure CSA and pure CSS. **B and C.** Principal component results (frist (Dim1) and second (Dim2) principal component) of plant accessions based on the expression levels of DEGs. **D and E.** GO and KEGG enrichments of DEGs. **F and G.** GO enrichment of CSA-ePAV and CSS-ePAV genes.


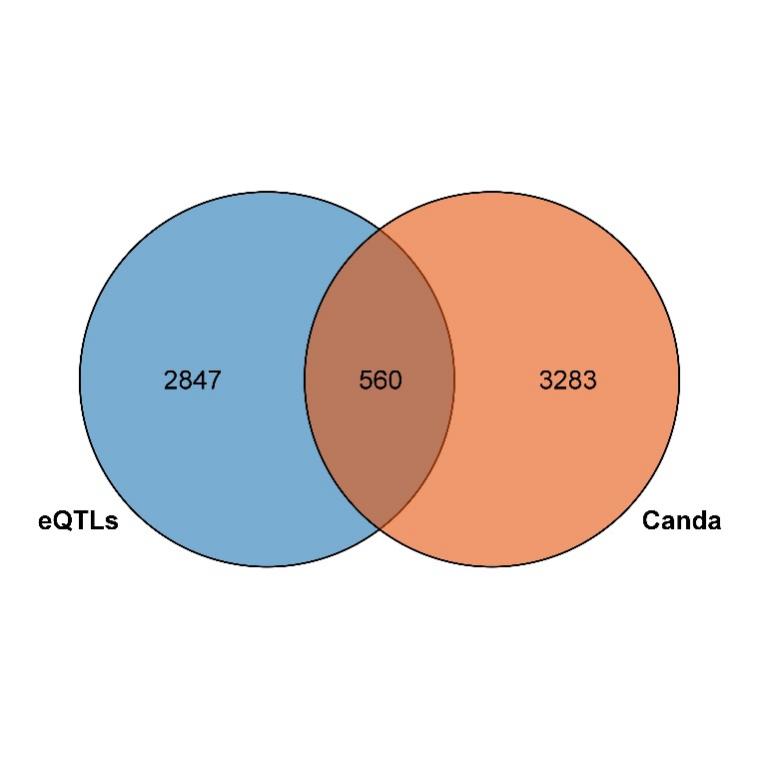


**Figure S5.** 560 genes are both candidate genes and eQTLs.


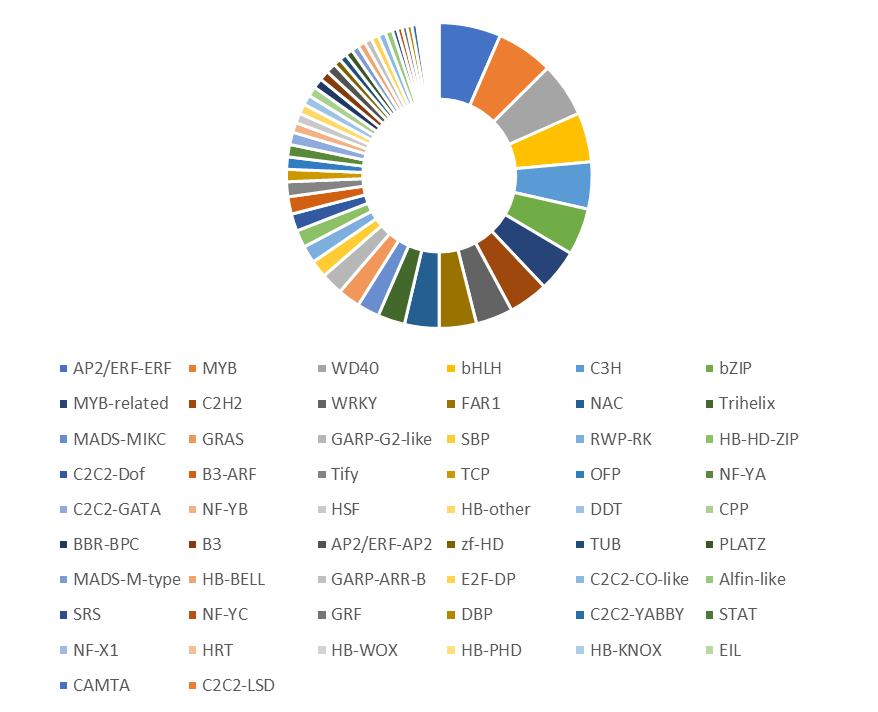


**Figure S6**. 382 TFs in the network of tea plant specialized metabolites


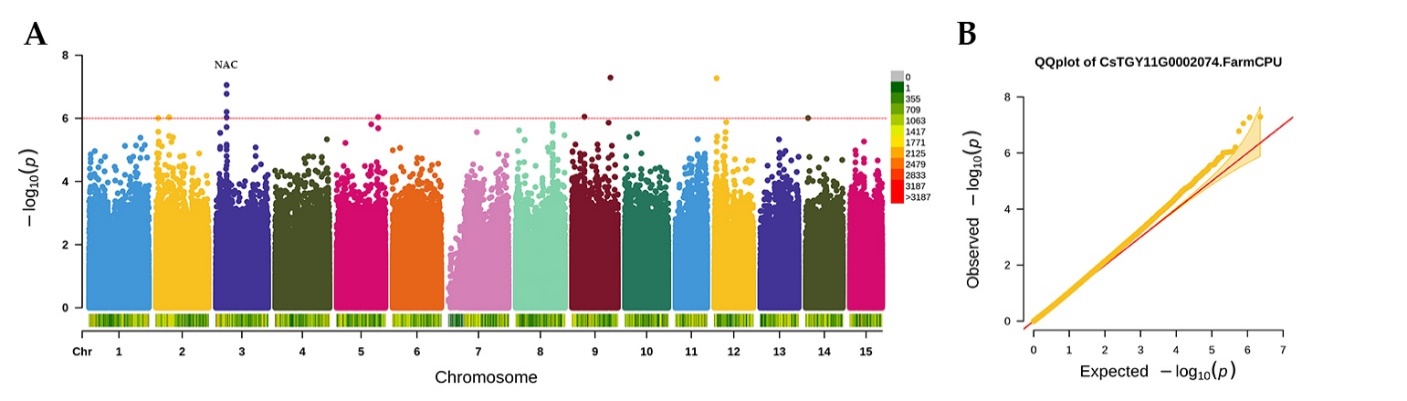


**Figure S7**. Manhattan plot (**A**) and QQ plot (**B**) of eQTLs of *CsTGY11G0002074* gene. The horizontal red line indicated the significance thresholds at –log10(1e-6).
