# Supplementary figures and images for: Pan-transcriptome assembly combined with multiple association analysis provides new insights into the regulatory network of specialized metabolites in the tea plant Camellia sinensis
Source: Hortic Res. 2022 Jul 2;9:uhac100. doi: 10.1093/hr/uhac100 (PMC9251601; doi:10.1093/hr/uhac100)

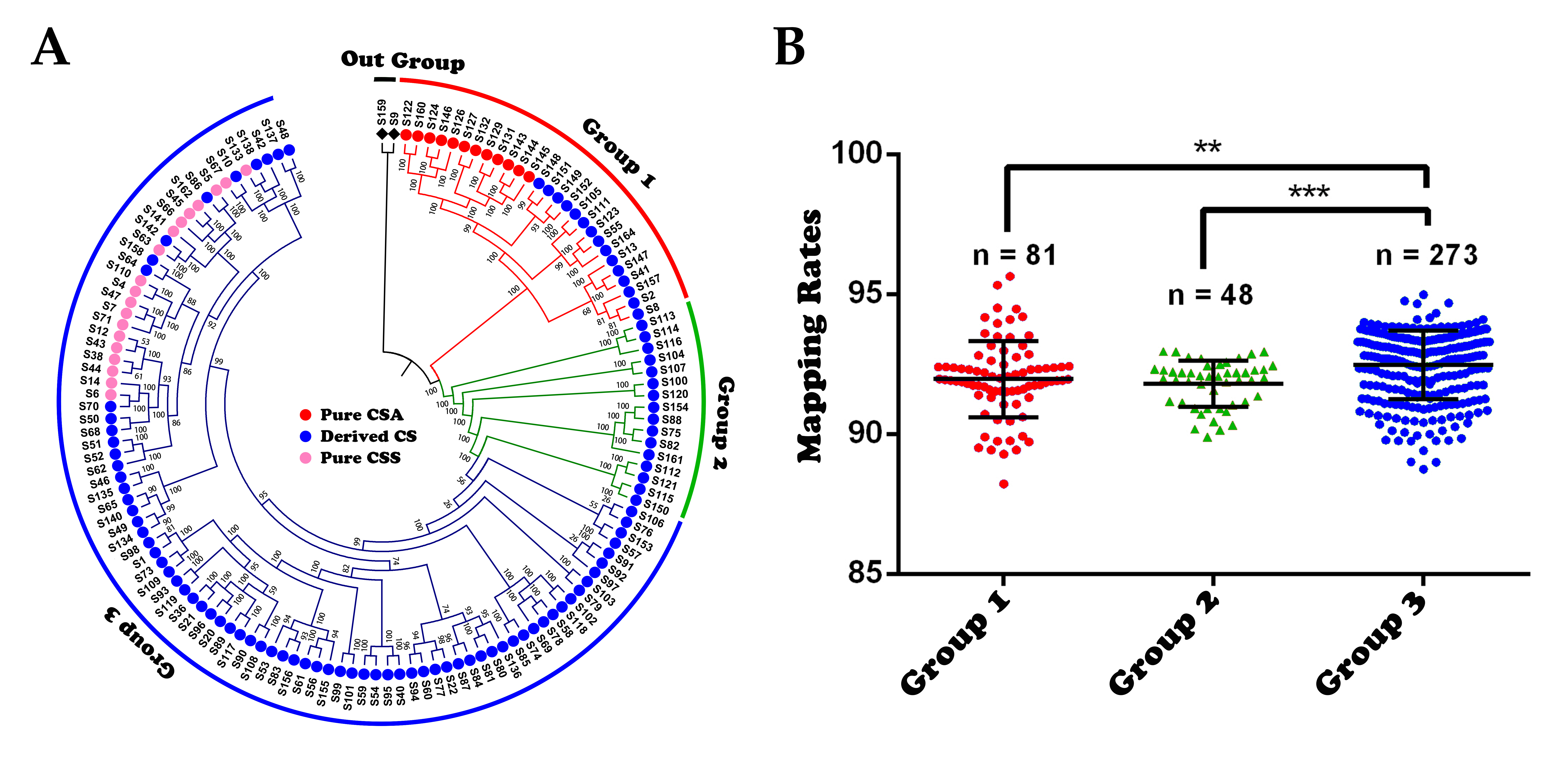

Supplement: Web_Material_uhac100 [file web_material_uhac100.zip › Figure-1.jpg]

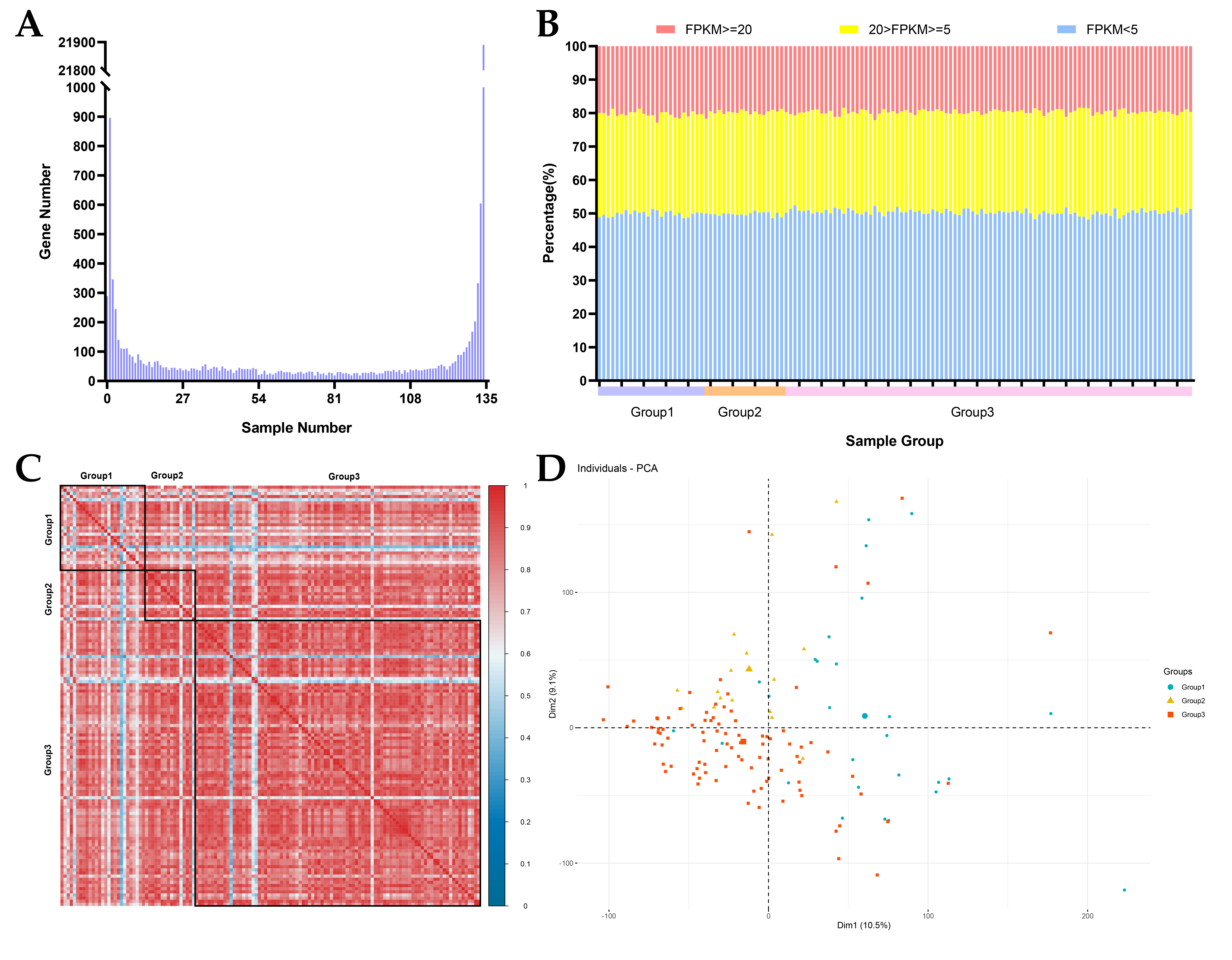

Supplement: Web_Material_uhac100 [file web_material_uhac100.zip › Figure-2.jpg]

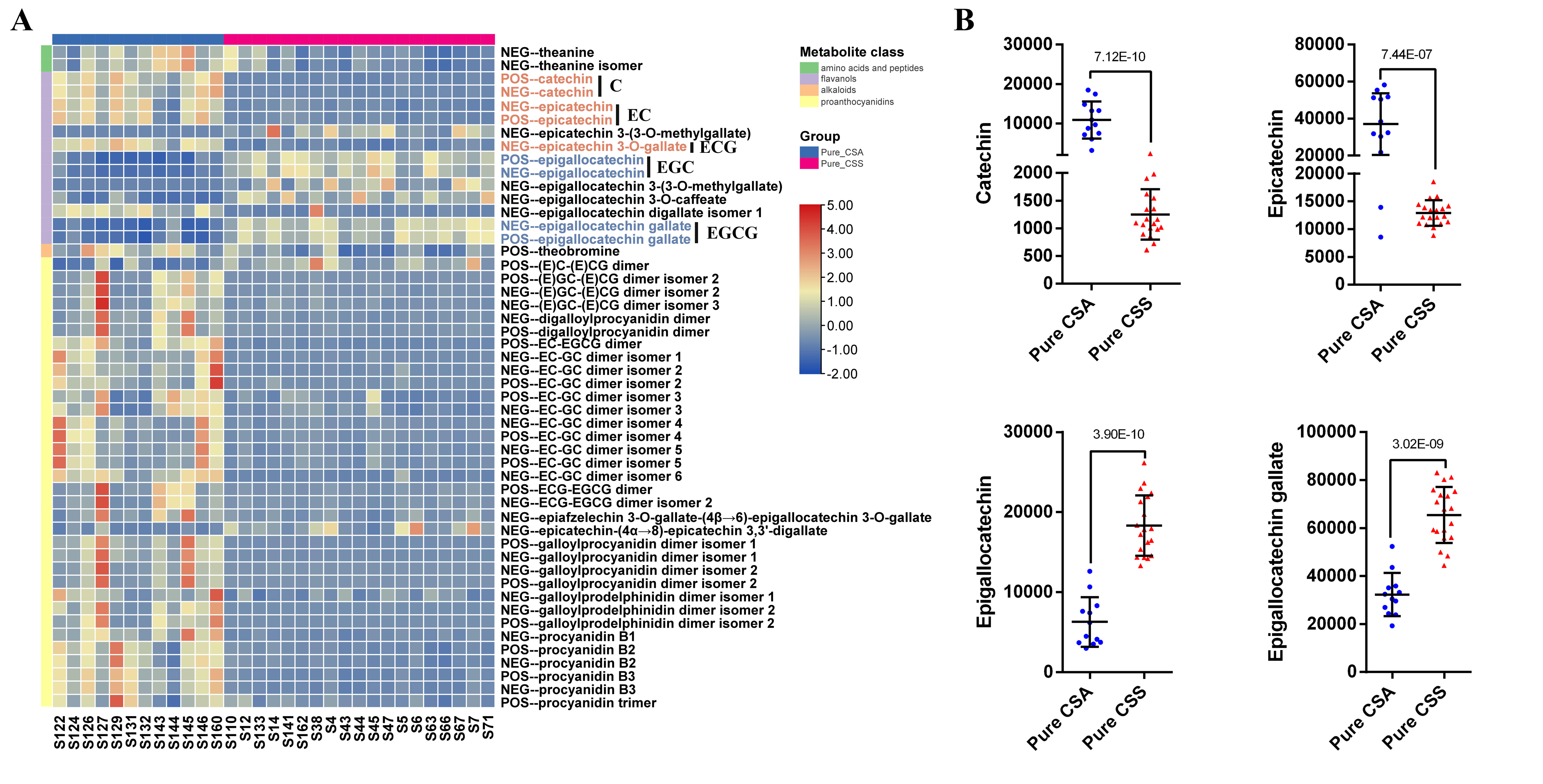

Supplement: Web_Material_uhac100 [file web_material_uhac100.zip › Figure-3.jpg]

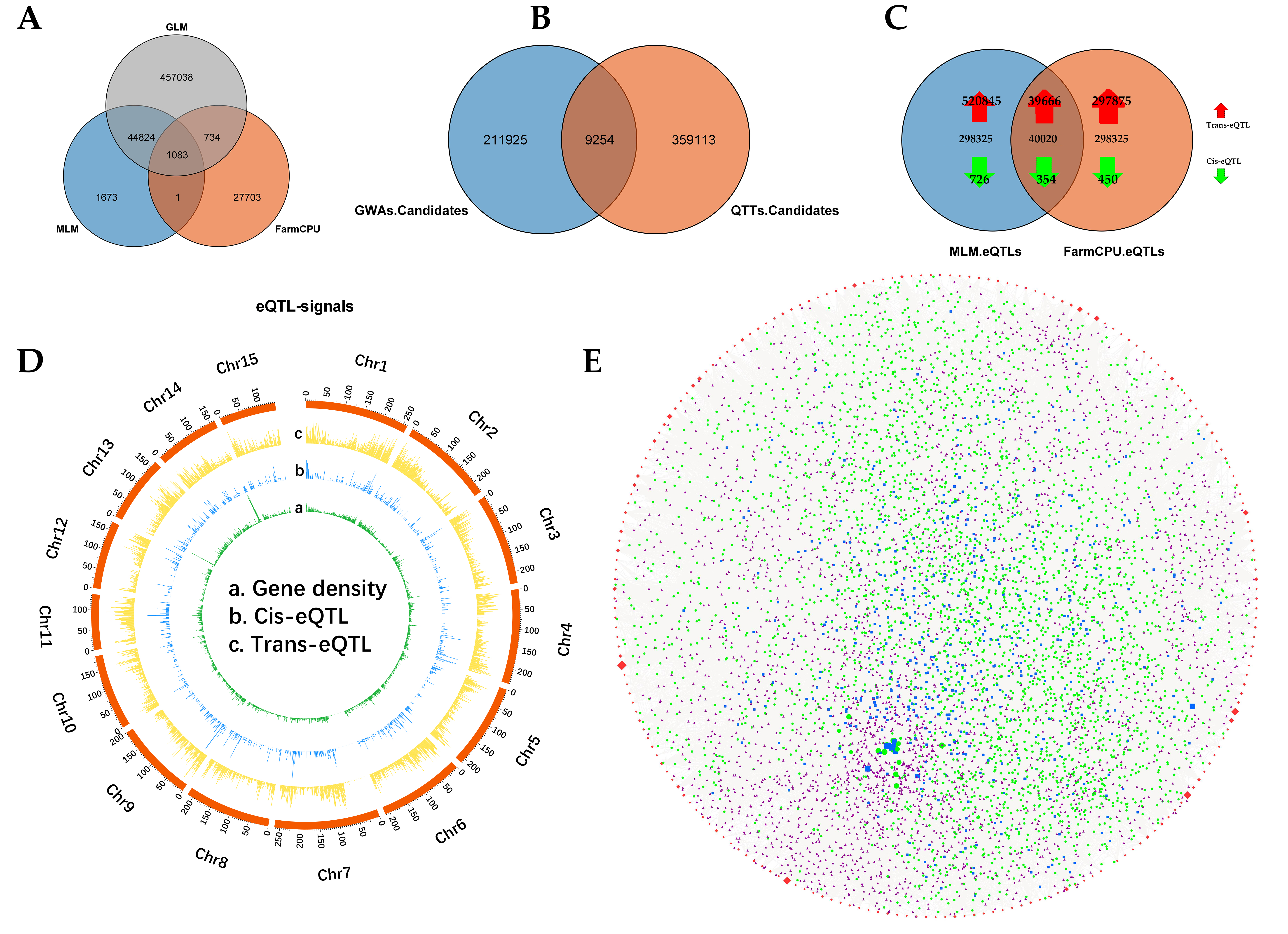

Supplement: Web_Material_uhac100 [file web_material_uhac100.zip › Figure-4.jpg]

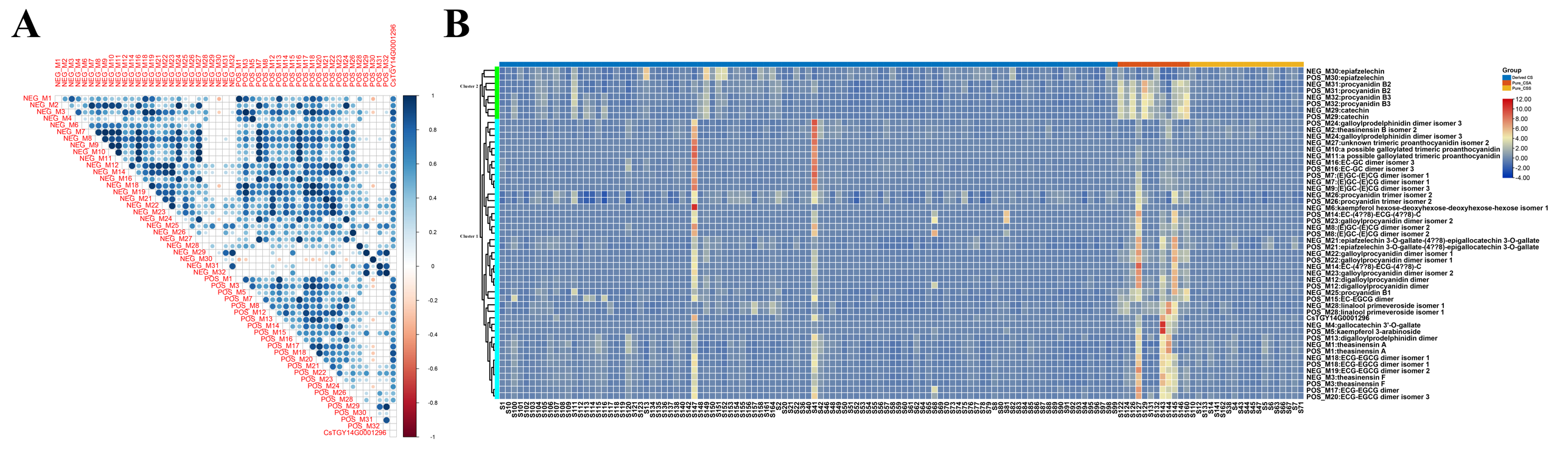

Supplement: Web_Material_uhac100 [file web_material_uhac100.zip › Figure-5.jpg]

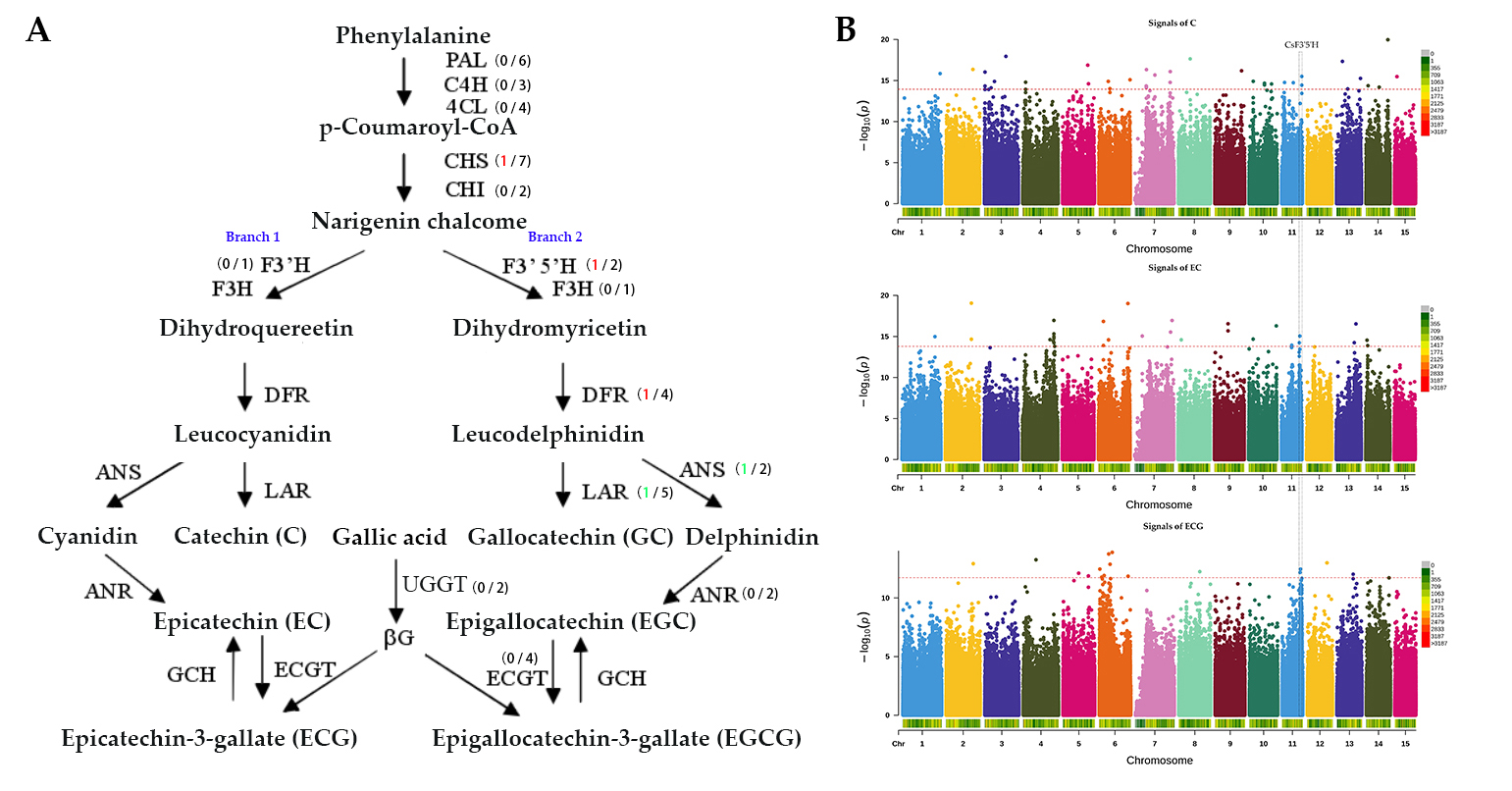

Supplement: Web_Material_uhac100 [file web_material_uhac100.zip › Figure-6.jpg]
